# Magnetic Resonance Imaging Confirmed Olfactory Bulb Reduction in Long COVID-19: Literature Review and Case Series

**DOI:** 10.3390/brainsci12040430

**Published:** 2022-03-24

**Authors:** Andrea Frosolini, Daniela Parrino, Cristoforo Fabbris, Francesco Fantin, Ingrid Inches, Sara Invitto, Giacomo Spinato, Cosimo De Filippis

**Affiliations:** 1 Department of Neuroscience DNS, University of Padova, 35100 Padova, Italy; francesco.fantin.3@studenti.unipd.it (F.F.); cosimo.defilippis@unipd.it (C.D.F.); 2 Audiology Unit, Treviso Hospital, 31100 Treviso, Italy; 3 Department of Otorhinolaryngology Head and Neck Surgery, ASST Sette Laghi, Ospedale di Circolo e Fondazione Macchi, 21100 Varese, Italy; daniela.parrino@gmail.com; 4 Otolaryngology Unit, Treviso Hospital, 31100 Treviso, Italy; cristoforo.fabbris@gmail.com; 5 Neuroradiology Unit, Treviso Hospital, 31100 Treviso, Italy; ingrid.inches@aulss2.veneto.it; 6 INSPIRE Lab, Department of Biological and Environmental Science and Technologies, DiSTeBA, University of Salento, 73100 Lecce, Italy; sara.invitto@unisalento.it

**Keywords:** olfactory perception, methodology for olfactory science, clinical aspects of olfaction, anosmia, magnetic resonance imaging, olfactory bulb

## Abstract

An altered sense of smell and taste was recognized as one of the most characteristic symptoms of coronavirus infection disease (COVID-19). Despite most patients experiencing a complete functional resolution, there is a 21.3% prevalence of persistent alteration at 12 months after infection. To date, magnetic resonance imaging (MRI) findings in these patients have been variable and not clearly defined. We aimed to clarify radiological alterations of olfactory pathways in patients with long COVID-19 characterized by olfactory dysfunction. A comprehensive review of the English literature was performed by analyzing relevant papers about this topic. A case series was presented: all patients underwent complete otorhinolaryngology evaluation including the Sniffin’ Sticks battery test. A previous diagnosis of SARS-CoV-2 infection was confirmed by positive swabs. The MRIs were acquired using a 3.0T MR scanner with a standardized protocol for olfactory tract analysis. Images were first analysed by a dedicated neuroradiologist and subsequently reviewed and compared with the previous available MRIs. The review of the literature retrieved 25 studies; most cases of olfactory dysfunction more than 3 months after SARS-CoV-2 infection showed olfactory bulb (OB) reduction. Patients in the personal case series had asymmetry and a reduction in the volume of the OB. This evidence was strengthened by the comparison with a previous MRI, where the OBs were normal. The results preliminarily confirmed OB reduction in cases of long COVID-19 with an altered sense of smell. Further studies are needed to clarify the epidemiology, pathophysiology and prognosis.

## 1. Introduction

An altered sense of smell and taste was quickly recognized to be one of the most characteristic symptoms among the neurological manifestations of severe acute respiratory syndrome coronavirus-2 (SARS-CoV-2) infection. [1]. The reported percentage of coronavirus disease 2019 (COVID-19) patients with olfactory dysfunction ranges from 41% to 48% according to recent meta-analyses [2,3]. Olfactory symptoms are relevant also due to their influence on quality of life, with alterations in appetite and weight changes [4]. Despite most patients experiencing a complete resolution of olfactory and gustatory dysfunctions, there is a 21.3% prevalence of a self-reported persistent altered sense of smell or taste 12 months after mild-to-moderate symptomatic COVID-19 [5]. The persistence of clinical symptoms of COVID-19 is a characteristic of post-acute COVID-19. The Center for Disease Control (CDC) has formulated “post-Covid conditions” to describe health issues that persist more than four weeks after being infected with SARS-CoV-2; these include the so-called “long Covid” [6].

To date, magnetic resonance imaging (MRI) findings of COVID-19-related smell alterations have been variable and not clearly defined. A recent systematic review of imaging studies in olfactory dysfunction secondary to COVID-19 concluded that hyperintensity in the olfactory bulb (OB), OB atrophy, cortical hyperintensity and hypometabolic cortical activity are findings at later stages of the disease, likely due to the direct neurotropism of SARS-CoV-2 [7,8].

With the aim of clarifying the radiological findings of persistent smell alterations that are COVID-19 related, we performed a literature review focusing on the OBs’ alterations in patients with clinically confirmed hyposmia post-COVID-19. Additionally, we report four cases of persistent hyposmia following COVID-19 in which a comparison with patients’ previous MRI imaging was available.

## 2. Materials and Methods

### 2.1. Literature Review

**Scheme 1:**
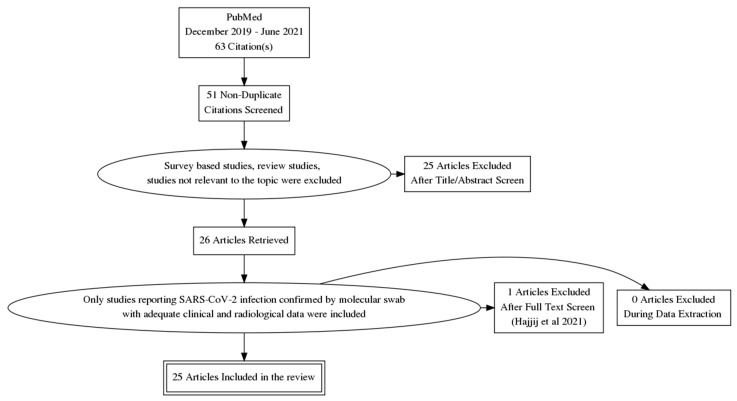
Flow-chart of literature research, from identification to inclusion of articles and patients.

A structured search of the English literature published on PubMed from 1 December 2019 to 30 June 2021 was conducted. The terms “MRI”, “Olfactory”, “Olfactory bulb” and “COVID-19” were used. Only inherent reports with SARS-CoV-2 positive patients, as confirmed with molecular nasopharyngeal swab, and with adequate clinical and diagnostic data were considered. Survey-based and cadaveric studies were excluded, as well as reviews, studies lacking data and studies clearly not related to the research. The PRISMA diagram (Scheme 1) summarizes the selection process.

### 2.2. Case Series

Data were examined in accordance with the Helsinki Declaration, the Italian privacy and sensitive data laws, and the in-house regulations of our hospital. All patients gave their written consent for clinical case publication.

Hyposmic and non-hyposmic patients with a post-SARS-CoV-2 brain–maxillofacial MRI and an available brain–maxillofacial MRI before infection were included in the present report. Hyposmic patients were considered as cases, whereas those who were non-hyposmic were considered as controls.

#### 2.2.1. Patients’ Management

All patients were evaluated at the Smell and Taste clinic of the Otolaryngology, Audiology and Phoniatrics Units, Department of Neuroscience Padua, Treviso Hospital between January and March 2021. Previous diagnosis of SARS-CoV-2 infection was confirmed by detection of the virus by polymerase chain reaction (PCR) on nasopharyngeal and throat swabs. All patients underwent complete Ear, Nose and Throat (ENT) evaluation including nasal endoscopy.

The *Sniffin’ Sticks* battery test was performed to evaluate olfactory function [9]. Results were interpreted according to the appropriate scoring system considering patients’ age. Scores were considered as “normal” (above 30 points), as “hyposmia” (between 30 and 15) and as “anosmia” (below 15).

Nasal washes with physiologic saline solution and nasal corticosteroid therapy (daily sprays of 100 mcg of mometasone furoate in each nostril, twenty days per month, as long as 4 months) were advocated together with olfactory rehabilitation exercises for the patients complaining of hyposmia [10]. A follow-up was set at the end of the prescribed treatment.

MRI scan was indicated in order to assess patients’ olfactory pathways related to persistent olfactory dysfunction.

#### 2.2.2. Magnetic Resonance Imaging

All MRI scans were first acquired and analysed by a dedicated neuroradiologist with a 15-year experience in the field. Subsequently, all images were reviewed and compared with the previous available MRIs.

The MRIs were acquired using a 3.0T MR scanner (Magnetom Vida, Siemens Healthcare, Erlangen, Germany) with a 64-channel head and neck coil with a standardized protocol for olfactory tract analysis. The protocol included axial T2W TSE covering the whole brain (matrix 512; FOV 230, 4 mm, TR 4090, TE 74), DWI (matrix 200, FOV 230, 4 mm, TR 2009, TE 66), coronal T2W covering the anterior and middle segments of the skull base (matrix 512, FOV 160, 2 mm, TR 7390, TE 80), cor T2 space isovolumetric (matrix 320, FOV 230, 0.7 mm, TR 1400, TE 158) and isotropic T1W MPR covering the whole brain (matrix 288, FOV 260, 0.9 mm, TR 2200, TE 2.53), isotropic FLAIR 3D (matrix 288, FOV 245, 0.9 mm, TR 8500, TE 386) and isotropic T1W MPR after gadolinium. All the 3D sequences were reconstructed in coronal, axial, sagittal projection for the radiologic evaluation. Intensity of OBs is defined as normal when bulbs have the same cortical intensity, as typically seen in healthy controls. Abnormal OBs’ intensity is defined when the bulb is more hyperintense than the cortex on T2WI and FLAIR. After gadolinium injection on T1WI, enhancement of the OBs is defined when they become more hyperintense in comparison with their intensity on pre-gadolinium T1WI [11].

Reduction in OB was diagnosed based on the following findings: flattening and thinning of the OB with loss of the normal oval shape, and an asymmetric decrease in the size of one OB compared with the contralateral side. OB volume was calculated by measuring the planimetric manual contouring of the OB obtaining the surface in mm^2^; after that, all the surfaces were added and multiplied by the thickness of the slices. Posterior end of the OB and beginning of the olfactory tract were determined when the measured surfaces of two successive slices were the same [12].

## 3. Results

### 3.1. Literature Review

After application of inclusion and exclusion criteria and full-text screening of retrieved data, 25 studies were included, with a total of 246 patients (116 females, 88 males, 42 not reported) [11,13,14,15,16,17,18,19,20,21,22,23,24,25,26,27,28,29,30,31,32,33,34,35,36]. Table 1 summarizes the main findings of the review. The included patients mainly experienced mild COVID-19 with sudden onset of hyposmia (or anosmia) and, in some cases, associated dysgeusia. Clinical Ear, Nose and Throat evaluation (ENT), when performed, consisted of endoscopy and olfactometry with the *Sniffin’ sticks* test [13,15,29,30], three-odorant Quick Smell Identification Test [17] or University of Pennsylvania Smell Identification Test (UPSIT) [30]. Seventeen out of 25 retrieved studies did not perform ENT evaluation and olfactometry [13,18,19,20,21,22,23,24,25,26,27,31,32,33,34,35,36]. 

The time that had elapsed from symptom onset to MRI evaluation ranged from 3 days [29] to 6 months [22]. The radiological evaluation was a brain MRI in the majority of cases. A brain computed tomography (CT) scan was performed in three studies [11,15,16] and positron emission tomography (PET) in another two [14,21]. Major MRI findings regarded the volume and signal intensity of the Olfactory Cleft (OC) and OB. A reduced volume and asymmetry of the OB was found in a total of 46 patients [11,15,18,23,24,33]. In the majority of these articles [18,23,24,33], the time between SARS-CoV-2 infection and the MRI was 4 weeks; one author [15] performed the MRI on the 15th day; another author did not report this data [11]. 

Normal OB volume was found among 71 patients [16,17,21,22,26,27,30,31,34,35,36]. A lots of these patients [16,17,21,27,30,35] underwent the MRI within the first month after infection; two authors performed the MRI after three [36] and six months [22], and three authors did not report the time elapsed between hyposmia and the MRI [26,31,34]. A case report revealed a transient augmented OB volume 7 days after SARS-CoV-2 infection that returned to a normal volume 24 days after [28]. Most of the studies [11,13,19,20,21,23,25,28,29,35] found hyperintensity of the OB in MRI scans performed in the first two months after SARS-CoV-2 infection.

The OC increased in volume or structural alterations in 72 patients [11,13,16,17,18,28]. Other relevant findings were the abnormality of the central olfactory pathway [14,15,22,29,30,33,34]. 

There was only one case report that included a comparison with an MRI undertaken previous to COVID-19 that confirmed a significant reduction in OB volume consistent with the reduction after infection [24].

### 3.2. Case Series

The chart review retrieved a total of five patients, who were included in the case series. They were all Caucasians and from the same country area (Veneto, Italy). All of them had pre- and post-infection MRIs. There were no significant demographic differences among the subjects.

#### 3.2.1. Case 1

**Figure 1:**
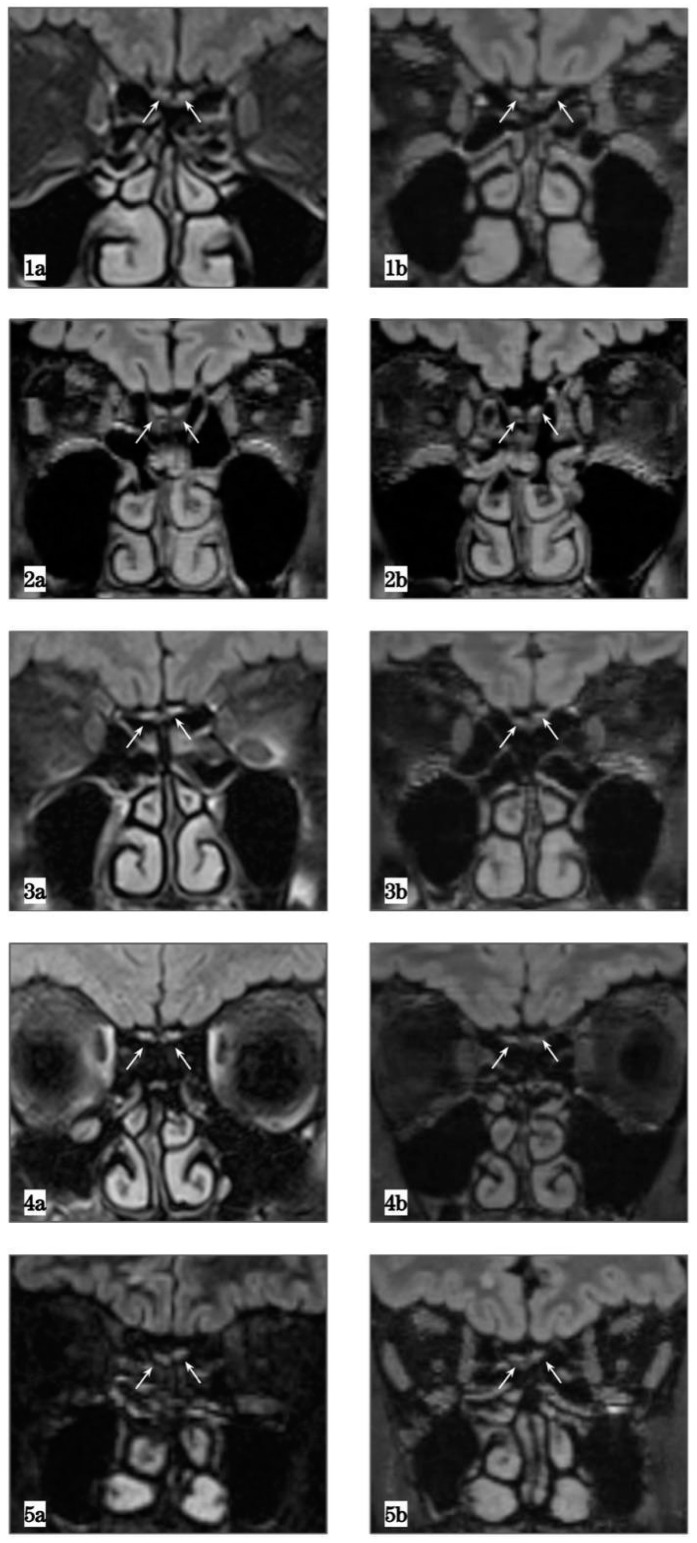
Magnetic resonance imaging (MRI) showing radiological findings of the included cases: four hyposmic patients (**1**–**4**) and one control (**5**). Frames (F.) (**1a**–**5a**) are pre-COVID-19 MRIs, showing normal olfactory bulb (OB) volumes. F (**1b**–**5b**) represent post-COVID-19 MRIs of the same patients revealing reduction in OB volume in hyposmic patients and unchanged OB in the subject without smell disorder (**5b**). All captures are isotropic FLAIR 3D (matrix 288, FOV 245, 0.9 mm, TR 8500, TE 386) sequences.

A 70-year-old man complained of persistent hyposmia and hypogeusia after SARS-CoV-2 infection that occurred in March 2020. He experienced a mild COVID-19 disease with 5 days of hospitalization. He denied having previous smell and taste disorders. Comorbidities were hypertension, hypercholesterolemia and atherosclerosis. The *Sniffin’ sticks* test resulted with a total score of 23/48 (subscales: 5 threshold, 9 discrimination, 9 identification) consistent with hyposmia. The patient had right deviation of the nasal septum, bilateral hypertrophy of the turbinates and minimal serous secretions, as observed with nasal endoscopy. Brain–maxillofacial MRI (performed in February 2021; Figure 1(1a)) showed bilateral OB reduction, with a flattened appearance of the upper profile, especially on the left side (size 26.07 mm^2^). No acute lesions were present. Comparison with a previous MRI (Figure 1(1b)), performed in October 2010, confirmed the normal OB volume (86.96 mm^2^ in the left size). During a telephone follow-up interview, one month after the test, the patient stated that his olfaction was slightly improving.

#### 3.2.2. Case 2 

A 70-year-old man reported unrecovered hyposmia and hypogeusia after SARS-CoV-2 infection. He tested positive in March 2020 and spent ten days home-isolating with a pauci-symptomatic course. No smell and taste disorders were present before infection. A total score of 24/48 (subscales: 5 threshold, 8 discrimination, 11 identification) resulted from the *Sniffin’ sticks* test, consistent with hyposmia. A complex deviation of the nasal septum partially obstructing the middle meatus was evident on nasal endoscopic examination. The patient underwent an MRI in February 2021: a discrete bilateral volume reduction was documented (44.94 mm^2^ right side, 37.24 mm^2^ left side; Figure 1(2b)) in comparison to the previous MRI, performed in December 2010, where the normal volume of the olfactory bulb previously to COVID-19 was detectable (81.36 mm^2^ right side, 77.42 mm^2^ left side; Figure 1(2a)). During a telephone follow-up interview, one month after the visit, the patient reported initial olfactory improvement.

#### 3.2.3. Case 3

A 59-year-old woman with persistent subjective hyposmia and dysgeusia 12 months after SARS-CoV-2 infection was evaluated. She was affected by moderate COVID-19 in March 2020. She did not have any pre-existent smell or taste disorders. She suffered from metabolic syndrome and vasculopathy. Her nasal endoscopy was normal. The *Sniffin’ sticks* test resulted in a total score of 32.5 (subscales: 10.5 threshold, 12 discrimination, 10 identification) consistent with normosmia.

An MRI showed a reduction in OB volume (Figure 1(3a,3b)), 19.44 mm^2^ on the right and 28.33 mm^2^ on the left side. The OB had homogeneous signal intensity (i.e., absence of active inflammation). The previous brain MRI (February 2016), showed double the OB volume (58.25 mm^2^ and 75.55 mm^2^ on the right and left side, respectively).

#### 3.2.4. Case 4

A 23-year-old woman with persistent subjective hyposmia 18 months after SARS-CoV-2 infection was evaluated. She did not have any pre-existent smell or taste disorders. Her nasal endoscopy showed a complex septal deviation and adenoid residue in nasopharynx. The Sniffin’ sticks test resulted in a total score of 30.5 (subscales: 4.5 threshold, 12 discrimination, 14 identification) consistent with normosmia.

An MRI showed a reduction in OB volume (Figure 1(4a,4b)), 45.93 mm^2^ on the right and 55.04 mm^2^ on the left side. The OB had homogeneous signal intensity (i.e., absence of active inflammation). The previous brain MRI performed eight years before showed normal OB volume.

#### 3.2.5. Case 5

A 45-year-old male patient was diagnosed with mild COVID-19 in March 2020. He did not complain of smell alterations and the otorhinolaryngology evaluation did not reveal any pathological findings (therefore, he did not undergo the Sniffin’ sticks test). Due to a persistent headache after infection, he underwent a brain MRI that revealed normal OB volume (66.87 mm^2^ right side and 48.3 mm^2^ left side). A similar volume had been observed in a previous MRI performed in 2019 (Figure 1(5a,5b)).

Results from the case series are summarized in Table 2.

## 4. Discussion

MRI findings in relation to COVID-19-linked olfactory dysfunction have not been systematically described to date. A recent literature review on the role of MRI in olfactory dysfunction showed that the establishment and validation of MRI-based biomarkers could help as a non-invasive method to be included in clinical practice in order to achieve a better diagnosis and treatment of olfactory loss [37]. Two possible mechanisms have been postulated for post-infectious hyposmia and parosmia: the peripheral hypothesis implies the incomplete regeneration of olfactory neurons, while the central hypothesis indicates dysfunction of the central olfactory pathway to the brain [22]. 

The first relevant result of our literature review was that the majority of retrieved studies (17 out of 25) only reported radiological findings without any data of clinical ENT evaluation and olfactometry [13,18,19,20,21,22,23,24,25,26,27,31,32,33,34,35,36]. Epidemiological and clinical characteristics were consistent with previously reported findings [1,5]. By considering all the retrieved patients, there was a slight female preponderance (56.86%, see Table 1); the typical age of onset was adult age and middle age (range 13 to 68, mean 39.33 years). Most patients experienced mild COVID-19 (89 patients), while fifteen patients had severe disease and one was asymptomatic; in the majority of patients (142), the COVID-19 classification was not reported [11,15,16,17,19,20,21,22,25,26,28,30]. Herein, we described the clinical and radiological findings of four patients with persistent subjective COVID-19-related hyposmia, who’s OB resulted in being atrophic (F. 1b,2b,3b,4b). This evidence was proven by comparison with a previous available MRI, where the OB was normal in size (F. 1a,2a,3a,4a). Additionally, we showed the case of a patient who did not suffer from smell and taste loss during SARS-CoV-2 infection. His post-COVID-19 MRI revealed normal OB volume, as found in the previous available MRI imaging (F. 5a,5b). The results of our cases are in accordance with what was retrieved in the review of the literature.

For several years, it has been proven that a reduced olfactory function is associated with decreased OB volumes in patients suffering from a wide range of diseases, including post-infectious olfactory disorder [38]. In addition, Yao et al. [39] showed that in patients with post-infectious olfactory loss, the OB volume is decreased and inversely correlated to the duration of symptoms. 

With regard to COVID-19 patients, reduction in the olfactory bulbs has been reported by many authors [11,15,18,23,24,33], and only in one case was it confirmed by comparison with previous serial MRIs in a patient with pituitary adenoma [24]. In cases of MRI performed early after SARS-CoV-2 infection (within two months after positive swab), signs of inflammation of the olfactory mucosa and OBs, such as hyperintensity on T2WI and FLAIR sequences, were also observed [11,13,19,20,21,23,25,28,29,35]. We did not notice hyperintense signals of the olfactory tract in our patients, probably due to the long period (11–18 months) between SARS-CoV-2 infection and MRI. In addition, according to a recent comparison of MRI findings among subjects with and without anosmia after COVID-19, no significant differences emerged [40]. The same conclusion was obtained in a large cohort study, by comparing longitudinal brain MRI changes among 713 subjects before and after SARS-CoV-2 infection [41].

Interestingly, Laurendon et al. [28] reported an increased volume of the OB and hyperintense signal in a single patient. This was defined as a transient OB edema because a follow-up MRI 24 days after the first evaluation showed normalization of the OB volume. Other authors [16,17,21,22,26,27,30,31,34,35,36] found a normal olfactory bulb volume in the early period after SARS-CoV-2 infection. Among these, Strauss et al. [21] stated that the presence of an increased T2 signal, reflecting the acuity of the time course from infection to MRI, could have played a role as a misleading factor. They hypothesize that if the individuals were scanned at longer-term follow-up, they would have observed volume loss in the absence of signal abnormality.

Several authors studied the central olfactory pathways in COVID-19 patients, reporting alterations in the central nervous system (e.g., olfactory tract, primary olfactory cortex, amygdala, temporal lobe) [14,15,22,29,30,33,34]. These authors found decreased metabolic activity without volume reduction in the tertiary olfactory cortex, which is involved in the quality processing and affective response to odorants. Lu et al. [14] found that recovered COVID-19 patients were more likely to have enlarged olfactory cortices, hippocampi, insulas, Heschl’s gyrus, Rolandic operculum and cingulate gyrus, and a decline in diffusion tensor imaging (DTI) values compared to controls. In our case series, we did not find any volumetric or signal alterations at the level of the central olfactory pathways.

After two years of pandemic and more than 400 million cases worldwide, the international community is now focusing on the study of the long-term implication of SARS-CoV-2 infection. The most common clinical symptoms in “long COVID” are fatigue, dyspnea, myalgia, cough, headache, joint pain, chest pain, altered smell, diarrhea and altered taste. In particular, alterations in smell have been reported in lots of cases of long COVID [42]. The main finding of our review is that post-COVID-19 with olfactory alteration, and possibly long COVID-19, could be characterized by OB reduction. Accordingly, we described our preliminary results of OB volume reduction in patients with clinically confirmed persistent hyposmia more than 3 months after SARS-CoV-2 infection. It is interesting to note how, in patients 3 and 4, the Sniffin’ sticks test was consistent with normosmia despite the OB reduction. The presence of olfactory memory could be implicated in the recovery process despite the anatomical alteration. The olfactory training, which aims to enhance olfactory function recovery exploiting the neuronal plasticity of the olfactory system, showed effectiveness in post-infectious olfactory dysfunction [43,44].

The main limitation of the present study is the low number of included patients in the case series. Therefore, data were heterogeneous. By considering the available data, the mean age of the patients retrieved from the literature review was 39.3 years (which is different from our case series, i.e., 53.4 years). Immunological reactions in a young adult may be quite different, compared to an older subject. For instance, the inflammation process would be more consistent, as well as the healing process, in a young patient. Therefore, further radiological analysis would allow even comparing imaging aspects of older patients to younger ones. Moreover, the gap between pre- and post-COVID-19 MRIs is quite large (8–11 years), leading to a possible discrepancy in anatomical features. It must be borne in mind that OB volumes may change with natural development or with olfactory training. Cases 1 and 2 previously underwent MRI in 2010, i.e., about 10 years before COVID-19. Their post-disease MRI revealed a consistent reduction in OB volume on both sides. Since the two patients were 70 years old, we cannot state that the radiological findings were due to the disease rather than the radio-anatomical evolution of OB features. Age alone, indeed, may determine reduction in the OB volume.

Due to these limitations, a higher number of cases would give more precise information, even in order to perform possible statistical analysis and to obtain more robust conclusions.

## 5. Conclusions

According to our results, olfactory bulb reduction could be a radiological sign in patients with long COVID-19 characterized by olfactory dysfunction. To the best of our knowledge, we reported the first series of patients with COVID-19-induced olfactory bulb reduction as confirmed by comparison with previous available MRI. There is the necessity for further studies, including meta-analysis of published data, to clarify radiological alterations and involvement of the peripheral and central olfactory pathways in patients with post-COVID-19 smell alterations in order to guide therapy and rehabilitation strategies.

## Figures and Tables

**Table 1:** Clinical characteristics and radiological findings of the patients from literature review.

Reference	Design	NoP (NoF)	Age	COVID-19 Classification	Time Symptoms–MRI	OB Size	OB Volume (mm^3^)	OB Hyperintensity	Other Findings
[11]	Perspective	23 (14)	29	NR	NR	Reduced	R:62; L:60.8	Yes	OC opacification
[13]	Perspective	23 (14)	39.0 ± 17.1	Mild illness	NR	NR	NR	Yes	OC edema
[14]	Perspective	60 (26)	44.10 ± 16.00	Mild illness (47 cases) Severe (13 cases)	90 days	NR	NR	NR	COP altered activity
[15]	Perspective	12 (10)	42	NR	15 days	Asymmetry	NR	No	OC edema; COP altered activity
[16]	Case-control	24 (14)	39.3 ± 12	NR	22 days	Normal	R: 59.76; L: 58.33	NR	OC reduced volume and inflammation
[17]	Case-control	20 (10)	34.6 ± 8.8	NR	6 days (1st MRI); 30 days (2nd MRI)	Normal	During infection: R: 37.7; L: 40.2 After infection: R: 38.1; L: 38.2	NR	OC obstruction (1st MRI)
[18]	Case-control	8 (6)	45.3	Mild Illness	70.5 days	Reduced	NR	NR	OC edema
[19]	Retrospective	5 (NR)	NR	NR	NR	NR	NR	Yes	NR
[20]	Retrospective	37 (NR)	NR	NR	NR	NR	NR	Yes	NR
[21]	Retrospective	12 (6)	58.25 ± 14.85	NR	<30 days	Normal	NR	Yes	NR
[22]	CR	1 (1)	28	NR	180 days	Normal	NR	No	COP altered activity
[23]	CR	1 (0)	21	Mild illness	28 days	Reduced	NR	Yes	NR
[24]	CR	1 (1)	19	Mild illness	60 days	Reduced	Before infection: R: 49.5; L: 47.46 After infection: R: 29.96; L: 35.51	NR	NR
[25]	CR	1 (1)	41	NR	14 days	NR	NR	Yes	NR
[26]	CR	1 (0)	27	NR	NR days	Normal	NR	No	NR
[27]	CR	3 (1)	13	Mild illness (2 cases) Severe (1 case)	<10 days	Normal	NR	No	NR
[28]	CR	1 (0)	27	NR	7 days	Augmented	R:64; L:73	Yes	OC edema
[29]	CR	1 (1)	25	Mild illness	3 days	NR	NR	Yes	COP altered activity
[30]	CR	5 (5)	NR	NR	<30 days	Normal	NR	No	COP altered activity
[31]	CR	1(0)	17	Mild illness	NR	Normal	NR	No	NR
[32]	CR	1(1)	60	Asymptomatic	During infection	NR	NR	No	NR
[33]	CR	1(1)	16	Mild illness	38 days	Asymmetry	NR	No	OT hyperintensity
[34]	CR	1(1)	68	Mild illness	NR	Normal	NR	No	OT hyperintensity
[35]	CR	2(2)	31	Mild illness (1 case) Severe (1 case)	25 days	Normal	NR	Yes	NR
[36]	CR	1(1)	25	Mild illness	90 days	Normal	NR	No	NR

Case report (CR), central olfactory pathway (COP), left side (L), olfactory bulb (OB), olfactory cleft (OC), olfactory tract (OT), magnetic resonance imaging of the olfactory pathways (MRI), number of patients (NoP), not reported (NR), right side (R), number of figure (NoF).

**Table 2:** Clinical characteristics of the included patients and comparison of olfactory bulb volumes (OBV), as measured in MRI after and before SARS-CoV-2 infection (F.Xn). Case 3 had moderate COVID-19, whereas all the others had mild COVID-19.

Case Number	Age, Gender	Sniffin’ Sticks Test	Before COVID-19 Left OBV (mm^3^)	After COVID-19 Left OBV (mm^3^)	Before COVID-19 Right OBV (mm^3^)	After COVID-19 Right OBV (mm^3^)
Case 1	70, M	23/48	86.96 (1a)	26.07 (1b)	69.4 (1a)	26.99 (1b)
Case 2	70, M	25/48	77.42 (2a)	37.24 (2b)	81.36 (2a)	44.94 (2b)
Case 3	59, F	32.5/48	75.55 (3a)	28.33 (3b)	58.25 (3a)	19.44 (3b)
Case 4	23, F	30.5/48	105.44 (4a)	55.04 (4b)	116.4 (4a)	45.93 (4b)
Case 5	45, M	NA	40.58 (5a)	48.3 (5b)	53.53 (5a)	66.87 (5b)

**Abbreviations**: olfactory bulb volume (OBV), not applicable (NA).

## Data Availability

The data are available on request to the corresponding author.
